# Pathogen‐inducible *Ta*‐*Lr34res* expression in heterologous barley confers disease resistance without negative pleiotropic effects

**DOI:** 10.1111/pbi.12765

**Published:** 2017-07-11

**Authors:** Rainer Boni, Harsh Chauhan, Goetz Hensel, Anne Roulin, Justine Sucher, Jochen Kumlehn, Susanne Brunner, Simon G. Krattinger, Beat Keller

**Affiliations:** ^1^ Department of Plant and Microbial Biology University of Zurich Zurich Switzerland; ^2^ Leibniz Institute of Plant Genetics and Crop Plant Research (IPK) Gatersleben Plant Reproductive Biology Seeland/OT Gatersleben Germany; ^3^ Plant Breeding Research Division Agroscope Zurich Switzerland; ^4^ Present address: Department of Biotechnology Indian Institute of Technology Roorkee Roorkee Uttarakhand 247667 India

**Keywords:** durable disease resistance, fungal pathogens, *Lr34/Yr18/Sr57/Pm38*, Barley, barley leaf rust, powdery mildew

## Abstract

Plant diseases are a serious threat to crop production. The informed use of naturally occurring disease resistance in plant breeding can greatly contribute to sustainably reduce yield losses caused by plant pathogens. The *Ta*‐*Lr34res* gene encodes an ABC transporter protein and confers partial, durable, and broad spectrum resistance against several fungal pathogens in wheat. Transgenic barley lines expressing *Ta*‐*Lr34res* showed enhanced resistance against powdery mildew and leaf rust of barley. While *Ta*‐*Lr34res* is only active at adult stage in wheat, *Ta*‐*Lr34res* was found to be highly expressed already at the seedling stage in transgenic barley resulting in severe negative effects on growth. Here, we expressed *Ta*‐*Lr34res* under the control of the pathogen‐inducible *Hv*‐*Ger4c* promoter in barley. Sixteen independent barley transformants showed strong resistance against leaf rust and powdery mildew. Infection assays and growth parameter measurements were performed under standard glasshouse and near‐field conditions using a convertible glasshouse. Two *Hv*‐*Ger4c::Ta‐Lr34res* transgenic events were analysed in detail. Plants of one transformation event had similar grain production compared to wild‐type under glasshouse and near‐field conditions. Our results showed that negative effects caused by constitutive high expression of *Ta*‐*Lr34res* driven by the endogenous wheat promoter in barley can be eliminated by inducible expression without compromising disease resistance. These data demonstrate that *Ta*‐*Lr34res* is agronomically useful in barley. We conclude that the generation of a large number of transformants in different barley cultivars followed by early field testing will allow identifying barley lines suitable for breeding.

## Introduction

To ensure global food security is one of the top challenges in this century. The ever‐growing world population is the main driver for increasing demand for agricultural products until 2050 (Gerland *et al*., 2014). Loss of agricultural land and climate change are additional factors that require a higher productivity (Godfray *et al*., 2010). Plant pathogens are responsible for worldwide crop losses of 10%–16% on average (Chakraborty and Newton, 2011; Oerke, 2006). A major strategy to improve the efficiency of crop production is to enhance plant resistance against diseases by taking advantage of the large diversity of naturally existing resistance genes. To do so, identified resistance genes can be transferred to other crop cultivars by classical breeding or introduced into other plant species by stable genetic transformation. Most resistance genes are specific to one pathosystem, but in a few cases resistance genes were shown to be functional in several plant–pathogen interactions. Maize *Rxo1* is an example of a resistance gene that was functionally transferred to the heterologous grass species rice where it mediates resistance against *Xanthomonas oryzae* pv. *oryzicola* (Zhao *et al*., 2005). *Pto*, a resistance gene originating from tomato and conferring resistance to *Pseudomonas syringae* pv. tomato, was functional against strains of *P.s*. pv. *tabaci* expressing the effector *avrPto* after stable transformation into *Nicotiana benthamiana* (Rommens *et al*., 1995).

An interesting group of durable, multi‐pathogen resistance genes against fungal pathogens has been specifically identified in hexaploid bread wheat (*Triticum aestivum*): It includes *Lr67* (=*Yr46/Sr55/Pm46*), *Lr46* (=*Yr29/Sr58/Pm39*) and *Lr34* (=*Yr18/Pm38/Sr57*). All these genes confer similar phenotypes in wheat, including a senescence‐like phenotype in the flag leaf described as leaf tip necrosis (LTN) (Singh and Huerta‐Espino, 1997) and partial disease resistance. *Lr67* and *Lr34* have been cloned and were found to encode a hexose transporter (Moore *et al*., 2015) and an ATP‐binding cassette (ABC) transporter, respectively (Krattinger *et al*., 2009). The LR34 and LR67 proteins are not classical resistance proteins such as receptor‐like kinases (RLKs), receptor‐like proteins (RLPs) and nucleotide‐binding, leucine‐rich repeat receptor proteins (NLRs) (Krattinger and Keller, 2016) and their molecular function is not yet fully understood. *Lr67*,* Lr34,* and *Lr46* were shown to confer partial resistance against leaf rust (*Puccinia triticina*), stripe rust (*P. striiformis* f.sp. *tritici*), stem rust (*P. graminis* f.sp. *tritici*), and powdery mildew (*Blumeria graminis* f.sp. *tritici, Bgt*) of wheat (Ellis *et al*., 2014; Spielmeyer *et al*., 2013).

*Ta*‐*Lr34* has been successfully used in wheat resistance breeding since the beginning of the last century (Kolmer *et al*., 2008). *Ta*‐*Lr34* has two predominant alleles in the wheat gene pool, referred to as *Ta*‐*Lr34res* (resistant) and *Ta*‐*Lr34sus* (susceptible) (Dakouri *et al*., 2010; Lagudah, 2011). *Ta*‐LR34res and *Ta*‐LR34sus differ only by two amino acid polymorphisms, and *Ta*‐*Lr34res* evolved from the ancestral *Ta*‐*Lr34sus* version after domestication (Krattinger *et al*., 2013). Orthologues of *Ta*‐*Lr34res* were detected in rice and sorghum, but not in maize and barley. Based on the two critical amino acid polymorphisms of *Ta*‐LR34res in wheat, all of these orthologues were identified as susceptible haplotypes (Krattinger *et al*., 2013, 2011).

*Ta*‐*Lr34res* was transformed to rice (Krattinger *et al*., 2016), barley (Risk *et al*., 2013), and maize (Sucher *et al*., 2016). In all three grass species, *Ta*‐LR34res was found to be functional: In rice it mediated resistance against rice blast (*Magnaporthe oryzae*), accompanied by a late LTN phenotype (Krattinger *et al*., 2016). In barley, *Ta*‐*Lr34res*, but not *Ta*‐*Lr34sus*, provided strong resistance against barley leaf rust (*Puccinia hordei*) and barley powdery mildew (*Blumeria graminis f. sp hordei, Bgh*). In contrast to wheat, *Ta*‐*Lr34res* under control of its native promoter was highly expressed already at seedling stage in barley, resulting in an early strong LTN phenotype. Thus, *Ta*‐*Lr34res* activity is accompanied by drastic pleiotropic effects with high fitness costs in barley.

Reducing or eliminating negative effects of a transgene can be achieved by using a different genetic background or by altering the expression level. As the negative effects of *Ta*‐*Lr34res* were described to be expression level‐dependent (Chauhan *et al*., 2015), we tested whether altered *Ta*‐*Lr34res* expression can result in barley plants with good levels of disease resistance but no negative effects on growth vigour. We hypothesize that to minimize negative effects on barley growth, *Ta*‐*Lr34res* should only be active when resistance is needed, i.e. at the early stage of infection. In this case, the promoter of choice would be pathogen‐inducible. Pathogen‐inducible promoters are activated early after infection (Hernandez‐Garcia and Finer, 2014). A well‐described pathogen‐induced barley (*Hordeum vulgare*) gene is *Hv*‐*Ger4c*, which belongs to a cluster of nine genes on barley chromosome 4H (Druka *et al*., 2002). Himmelbach *et al*. (2010) fused the *Hv*‐*Ger4c* promoter to GUS and performed transient expression assays as well as experiments using stably transformed barley plants. Plants inoculated with *Bgh* and *Puccinia hordei* as well as the non‐host pathogens *Bgt* and *Puccinia triticina* showed increased expression levels compared to non‐infected controls, regardless if the interaction was compatible or not. Expression was measured in epidermal peels as well as whole leaf tissue. The results revealed an epidermis‐specific expression pattern of the *Hv*‐*Ger4c* promoter. Further, Himmelbach *et al*. (2010) reported that *Hv*‐*Ger4c* is not activated upon abiotic stress treatment such as ozone, cold, wounding, UV irradiation or wind. This made the *Hv*‐*Ger4c* promoter an excellent candidate for our study. Here, we provide proof of concept that induced, epidermis‐specific expression of *Ta*‐*Lr34res* in barley results in broad spectrum resistance without compromising fitness and this can provide much needed inbuilt resistance against fungal pathogens.

## Results

### Characterization of the barley lines transgenic for *Hv*‐*Ger4c*::*Ta*‐*Lr34res*

We stably transformed barley cv. Golden Promise with *Ta*‐*Lr34res* under the control of the *Hv*‐*Ger4c* promoter and characterized the resulting transgenic lines (later called *Hv*‐*Ger4c*::*Ta‐Lr34res* lines) for transgene expression, disease resistance, and growth parameters. Nineteen primary transformants (T0) were obtained and grown to maturity. Eighteen T0 plants produced grains and T1 plants were genotyped by PCR. Plants were analysed regarding the development of LTN, resistance to the barley powdery mildew isolate K1 and the expression of the full‐length *Ta*‐*Lr34res* coding sequence (cDNA) (Table S1).

To identify potential transformants with only slightly reduced or unaffected fitness but good disease resistance, 2‐week‐old T1 plants were analysed in a first step for the presence/absence of LTN. In addition, resistance to powdery mildew was analysed on 3‐week‐old plants 7 days after infection (dpi). Progeny of sixteen events showed either strong LTN at seedling stage (six T1 progeny, Table S1) similar to line BG 9 with the native *Ta*‐*Lr34res* promoter (Chauhan *et al*., 2015; Risk *et al*., 2013) or were susceptible (eight T1 progeny) and were therefore not of interest for further investigation. T1 progeny of events 8 and 11 showed no or reduced LTN compared to line BG 9. Both lines showed resistance to powdery mildew 7 dpi at a comparable level as line BG 9. For further analyses, progeny of events 8 and 11 was selected for uniform resistance to powdery mildew in T2. To do so, at least 24 T2 seedlings of different individual T1 plants were grown to 2‐week‐old plantlets and infected with barley powdery mildew. T2 families showing uniform resistance and no segregation pattern in the genotype based on PCR were considered as uniform lines. Southern blot analysis (based on a probe derived from the marker gene used in transformation) of these T2 lines, uniform for resistance and LTN phenotype, detected three non‐segregating insertions and one segregating insertion in line 8 and two insertions of which one segregated in line 11 (Figure S1). In both lines 8 and 11, the segregating insertion had no influence on the resistance phenotype, indicating that uniform lines are uniform for the functional insertion(s) represented by the non‐segregating bands in the Southern blot (Figure S1). Therefore, seeds of uniform lines were pooled within transformation events and used for further investigation. Similarly, the corresponding azygous sister lines (null‐segregants) were determined.

### *Hv*‐*Ger4c*::*Ta‐Lr34res* lines showed enhanced resistance to barley leaf rust and powdery mildew

**Figure 1:**
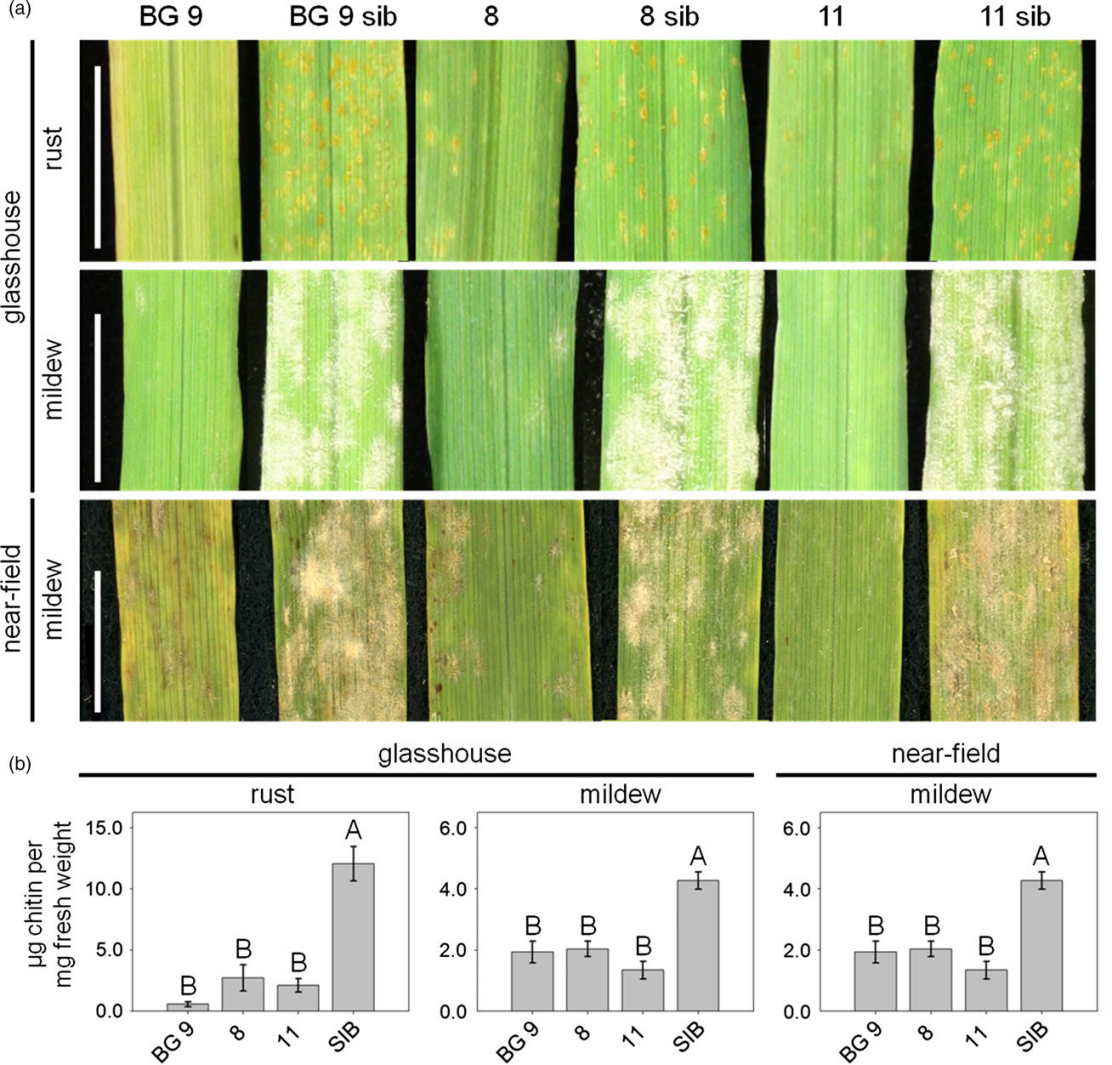
*Hv*‐*Ger4c*::*Ta*‐*Lr34res* transgenic barley shows *Ta*‐*Lr34res*‐mediated disease resistance. For assessment of *Ta*‐*Lr34res*‐mediated resistance under standard glasshouse conditions, the fourth leaves of plants at the five‐leaf stage were taken 7 dpi. For the assessment of resistance under near‐field conditions, fourth leaves of six‐leaf stage plants were evaluated. Leaves were used for macroscopic observations (a) as well as chitin measurement (b). Columns show the amount of chitin in μg chitin per mg fresh weight as an average of 3–7 biological replicates. Samples of all azygous sister lines were pooled. Error bars indicate standard errors. Scale bars represent 1 cm. Statistical analysis was performed on square‐root‐transformed (rust, glasshouse) or non‐transformed (near‐field) values using the all pairs Tukey–Kramer HSD test. In case of mildew, glasshouse, Kruskal–Wallis test on non‐transformed values was performed. Levels not connected by the same letter are significantly different (*P*‐values: <0.02).

To further characterize pathogen resistance, fourth leaves of T2 plants of the lines 8, 11, BG 9 and their corresponding azygous sister lines were taken for macroscopic and biochemical analyses of *Ta*‐*Lr34res*‐mediated resistance. We performed infection assays with the barley leaf rust isolate BRG1.2.1 and the barley powdery mildew isolate K1 as described in Risk *et al*. (2013). Line BG 9 developed no visual symptoms upon infection with barley leaf rust, while the corresponding azygous sister line BG 9 sib was fully covered by uredia (Figure 1a). In line 8, a much lower amount of uredia developed compared to the azygous sister line 8 sib. In addition, uredia were smaller and surrounded by chlorotic flecks (Figure 1a), mimicking the partial resistance in wheat. In line 11, mainly chlorotic spots and a few very small uredia were observed, whereas the corresponding azygous sister line 11 sib was fully covered by uredia, indicating full susceptibility (Figure 1a). In the infection assay using barley powdery mildew, line BG 9 showed only a few chlorotic spots and both lines 8 and 11 developed only a few mildew pustules, while all azygous sister lines were completely covered by the fungus (Figure 1a). To assess whether resistance of the barley lines transgenic for *Ta*‐*Lr34res* under the control of the *Hv*‐*Ger4c* promoter might be of interest for agricultural application, an infection assay was performed under near‐field conditions. To do so, the same lines tested in the glasshouse were grown in a convertible glasshouse (Romeis *et al*., 2007) and artificially infected with barley powdery mildew. Under these near‐field conditions, similar macroscopical results were observed regarding resistance to powdery mildew as recorded in the glasshouse, indicating the functionality of the *Hv*‐*Ger4c*::*Ta*‐*Lr34res*‐mediated resistance under near‐field conditions (Figure 1a). To quantify *Ta*‐*Lr34res*‐mediated resistance, the amount of the fungal cell wall component chitin was measured using the biochemical WGA‐FITC method (Ayliffe *et al*., 2014, 2013). The fourth leaves of plants at the five‐leaf stage were taken in the glasshouse experiment, whereas for the experiment under near‐field conditions fourth leaves at the six‐leaf stage were used for chitin measurements.

In the infection assay in the glasshouse using barley leaf rust, a Kruskal–Wallis test analysis indicates that overall differences in the level of chitin exist among lines (*X* ^2^(3, *N* = 42) = 31.2, *P* < 0.001). Post hoc comparison tests showed that there was a significantly reduced chitin amount in all *Hv*‐*Ger4c*::*Ta*‐*Lr34res* lines compared to the pooled azygous sister lines (all pair‐wise *P*‐values between transgenic lines and the azygous sister lines <0.008, Figure 1b). Further, chitin levels of lines 8 and 11 were not different from the one in line BG 9 (pair‐wise *P*‐values >0.5, Figure 1b). After infection with barley powdery mildew in the glasshouse, a Kruskal–Wallis test analysis revealed overall differences among lines (*X* ^2^(3, *N* = 42) = 29.8, *P* < 0.0001). Here again, post hoc pair‐wise comparison tests revealed that chitin amounts were clearly reduced compared to the pooled azygous sister lines (all pair‐wise *P*‐values <0.02) and chitin amounts of lines 8 and 11 were similar to the one in BG 9 (all pair‐wise *P*‐values >0.5).

Under near‐field conditions (Kruskal–Wallis test, (*X* ^2^(3,*N* = 42) = 26.5, *P* < 0.0001), all *Hv*‐*Ger4c*::*Ta*‐*Lr34res* lines showed a significant reduction in chitin amounts compared to the azygous sister lines (all pair‐wise *P*‐values <0.02) and reached similar chitin levels as the highly expressing line BG 9 (all pair‐wise *P*‐values >0.9, Figure 1b). Taken together, these results show that all *Hv*‐*Ger4c*::*Ta*‐*Lr34res* lines reach similar resistance levels as the highly expressing line BG 9 where the *Ta*‐*Lr34res* gene is under control of its own promoter.

### *Hv*‐*Ger4c*::*Ta*‐*Lr34res* lines show reduced LTN and reduced impact on growth parameters

**Figure 2:**
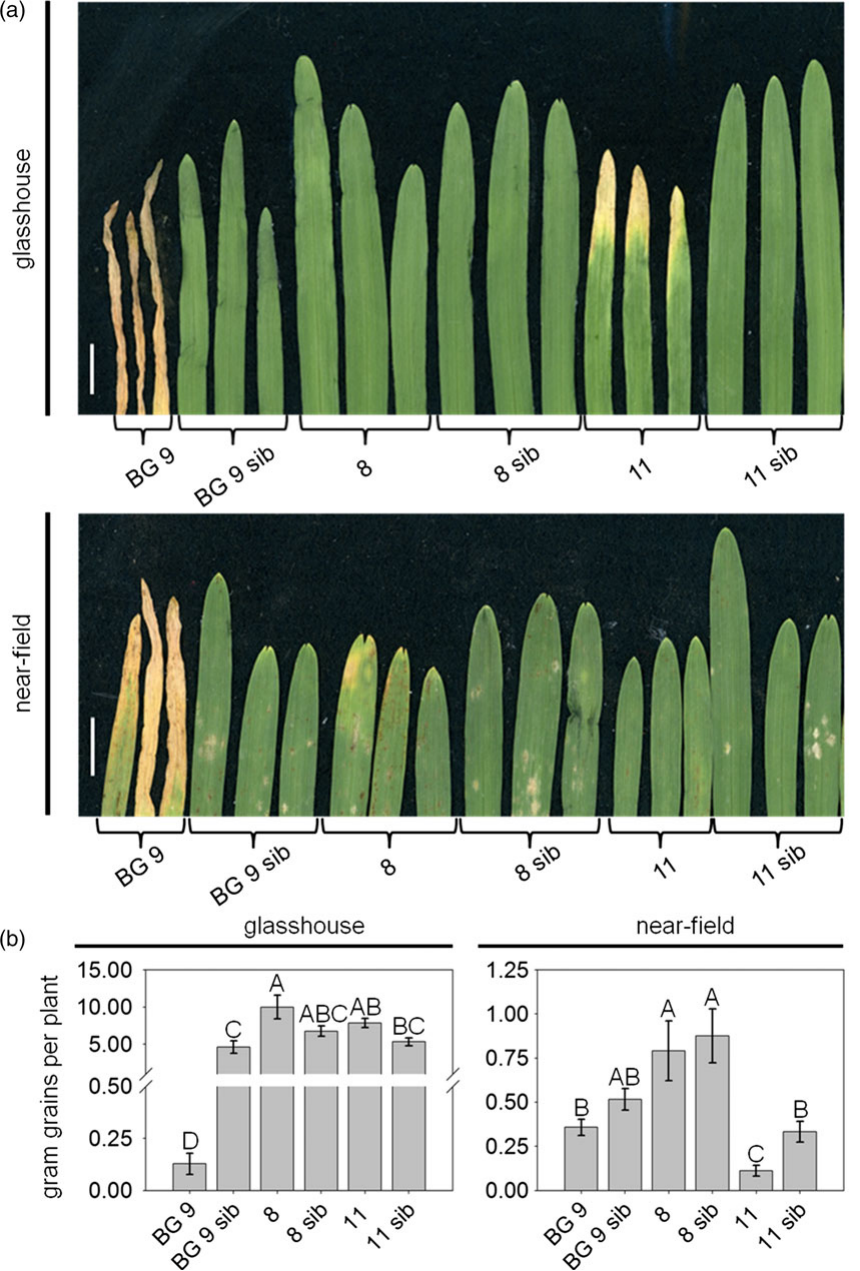
*Hv*‐*Ger4c*::*Ta‐Lr34res* barley lines show reduced LTN and reduced impact on growth parameters. (a) For the assessment of LTN, the first leaf of plants at the five‐leaf stage was taken. (b) For assessment of growth parameters, plants were grown until maturity, 10 individual plants were harvested and gram grains per plant were determined. Plants under standard glasshouse conditions were 138 days, plants under near‐field conditions were 140 days old. Scale bars represent 1 cm. Error bars indicate standard errors. Statistical analysis was performed on square‐root‐transformed values using the all pairs Tukey–Kramer HSD test. Levels not connected by the same letter are significantly different (*P*‐values: <0.05).

To investigate whether the inducible *Hv*‐*Ger4c*::*Ta*‐*Lr34res* lines show less negative effects on growth parameters compared to BG 9, seedlings were investigated for the presence and intensity of LTN. Whereas line 8 showed no LTN in the first leaf at the five‐leaf stage, line 11 showed LTN, but at a reduced level compared to BG 9 under glasshouse conditions (Figure 2a). The reduced LTN level at the five‐leaf stage might indicate an overall reduction in fitness costs. To determine possible negative effects of *Hv*‐*Ger4c*:: *Ta*‐*Lr34res* at later growth stages, plants were grown until maturity and growth parameters were measured. Growth parameters included gram of grains per plant, dry weight, number of spikes per plant, number of grains per spike, grains per plant and the 1000‐grain weight. To test the influence of environmental conditions on growth parameters, data were collected from plants grown in the glasshouse and under near‐field conditions, respectively (Figure 2 and Figure S2).

Under glasshouse conditions, lines 8 and 11 reached similar values in gram of grains per plant as their corresponding azygous sister lines (ANOVA (F(5,54) = 41.6, *P*‐value <2e^−16^), all pair‐wise *P*‐values >0.2, Figure 2b). Furthermore, for both lines similar values were measured compared to their azygous sister lines for all growth parameters tested (Figure S2 and Table S2). Lines 8 and 11 showed considerably higher values than BG 9 in all measurements, indicating an improvement of growth vigour by the use of the *Hv*‐*Ger4c* promoter to control *Ta*‐*Lr34res* expression (Figure 2b and Figure S2).

We also wanted to test whether altered expression of *Ta*‐*Lr34res* by the *Hv*‐*Ger4c* promoter would lead to improved growth vigour under near‐field conditions. All *Hv*‐*Ger4c*::*Ta*‐*Lr34res* lines showed no LTN phenotype compared to BG 9 at the seedling stage (Figure 2a). Line 8 performed the same as the azygous sister line for all growth parameters, indicating that *Ta*‐*Lr34res* was not linked to negative effects in this particular line (Figure 2b, Figure S2 and Table S2). At later stages, the negative effects on growth developed continuously in lines BG 9, 11 and 11 sib, resulting in lower values for gram grains (Figure 2), number of spikes per plant and grains per plant (Figure S2). Lines BG 9, 11 and 11 sib produced less dry weight biomass than line 8, line 8 sib and line BG 9 sib. This resulted in a lower number of spikes per plant and consequently a lower amount of grains per plant (Figure S2 and Table S2). In addition, lines BG 9, 11 and 11 sib lodged at the flowering stage. They produced grains on ground‐lying tillers, which lead to 1000‐grain weights which were mostly not different compared to their corresponding azygous sister lines (Figure S2 and Table S2). Because of the broken tillers, lines BG 9, 11 and 11 sib turned out not to have appropriate growth vigour under near‐field conditions. In most cases, growth values of line 11 did not differ from line 11 sib. This indicates that the negative effects observed for this line under near‐field conditions must have been caused by somaclonal or epigenetic variation due to the tissue culture process rather than by the presence of *Ta*‐*Lr34res*.

### Induction of *Ta*‐*Lr34res* expression by the *Hv*‐*Ger4c* promoter

**Figure 3:**
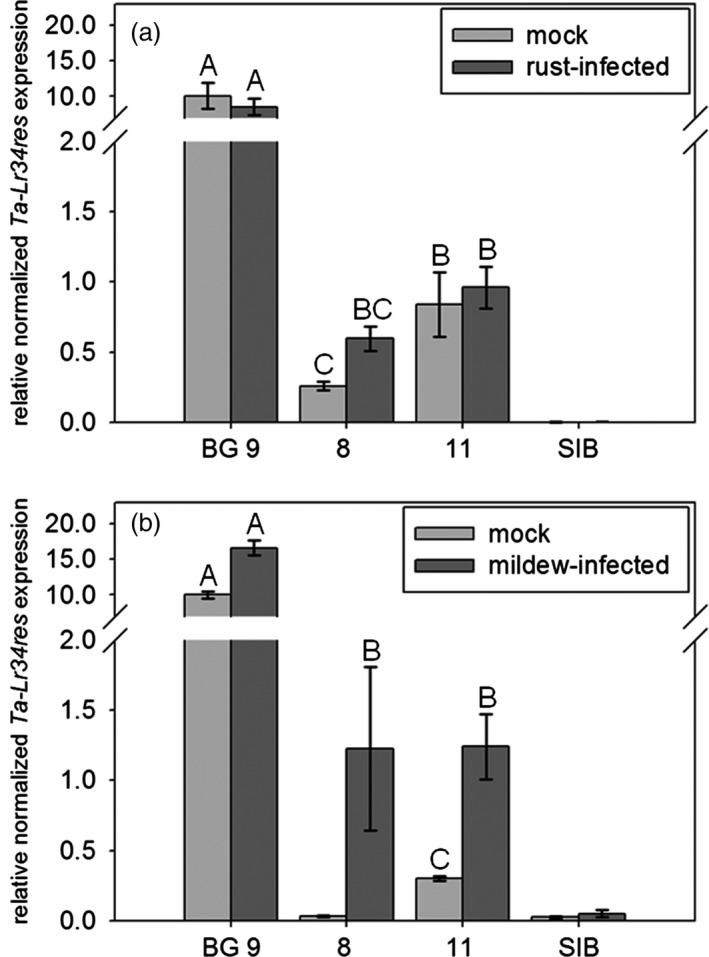
Expression analysis of *Ta*‐*Lr34res*. The second leaf of nineteen‐day‐old plants, 9 dpi rust (a) and powdery mildew (b), were harvested for RNA extraction and expression analysis by RT‐qPCR. Bars show the relative *Ta*‐*Lr34res* expression normalized to *GAPDH* as an average of 4–7 biological replicates. Basal expression level of line 8 was below detection level in the mildew experiment possibly due to different growth conditions between the rust and mildew infection experiment. SIB represents the average of all azygous sister lines representing the negative control. Error bars represent standard errors. Statistical analysis was performed on log_10_‐transformed expression values using the all pairs Tukey–Kramer HSD test. Levels not connected by the same letter are significantly different (*P*‐value: <0.001).

ANOVA analysis of expression data revealed significant differences of *Ta*‐*Lr34res* expression among the tested lines (rust glasshouse: (F(5,32) = 47.9, *P*‐value <0.0001, Figure 3a), mildew glasshouse: (F(4,28) = 93.2, *P*‐value <0.0001, Figure 3b) near‐field: (F(2,18) = 42.6, *P*‐value <0.0001, Figure S4). Expression analysis revealed that the *Ta*‐*Lr34res* expression levels of the uninfected *Hv*‐*Ger4c*::*Ta*‐*Lr34res* lines were 12‐ to 39‐fold lower compared to the uninfected line BG 9 carrying the *Ta*‐*Lr34res* promoter (Figure 3) under glasshouse conditions. A low level of basal expression in uninfected plants of line 8 was detectable by RT‐qPCR in the rust assay but not in the mildew experiment. This is possibly due to the different plant growth conditions used in the mildew and rust experiments. Expression levels under near‐field conditions of uninfected *Hv*‐*Ger4c*::*Ta*‐*Lr34res* plants were 5‐ to 11‐fold lower compared to BG 9 (Figure S4).

To assess whether *Ta*‐*Lr34res* expression was pathogen‐inducible after fusion to the *Hv*‐*Ger4c* promoter, infection assays with powdery mildew and barley leaf rust were performed. Fourteen‐day‐old seedlings were inoculated with barley powdery mildew and barley leaf rust, respectively. After 7 dpi (mildew) and 10 dpi (rust), third leaves were harvested for expression analysis. Infected leaves were compared to mock‐infected leaves grown at the same time and under the same conditions. Interestingly, no induction upon rust infection was detected (all pair‐wise comparison between mock‐infected and infected lines *P*‐values >0.7, Figure 3a). In contrast, *Ta*‐*Lr34res* expression was induced upon infection with powdery mildew in all *Hv*‐*Ger4c*::*Ta*‐*Lr34res* lines (line 11: pair‐wise *P*‐value <0.0001). Expression in line 11 was fourfold higher upon infection. In line 8, the expression level upon mock infection was below detection level and therefore no statistical analysis was possible. Yet, expression of *Ta‐Lr34res* was detectable in infected line 8 and reached similar expression levels as infected line 11 (pair‐wise comparison *P*‐value = 0.86), indicating that *Ta*‐Lr34res was also induced upon infection in line 8 (Figure 3b).

## Discussion

Enhancing resistance by alteration of expression levels is frequently accompanied by increased fitness costs. The over‐expression of tomato *Pto* (Tang *et al*., 1999) or of the NLR gene *BAL* in *Arabidopsis* (Stokes *et al*., 2002) led to microscopic lesions or dwarfing and twisted leaves, respectively. Furthermore, wheat lines over‐expressing the NLR gene *Lr10* showed reduced grain weight compared to the *Lr10* donor line under near‐field conditions (Feuillet *et al*., 2003; Romeis *et al*., 2007). Interestingly, different pleiotropic effects caused by the same transgene have been observed among different transformation events. In one of four transgenic lines over‐expressing the wheat powdery mildew disease resistance gene *Pm3b*, no pleiotropic effects were observed under field conditions. In the three lines Pm3b#2‐4, plants showed different level of chlorosis and reduced fertility, whereas this phenomenon was not observed in line Pm3b#1 (Brunner *et al*., 2011). The different phenotypes of the two genotypes Pm3b#2 and Pm3b#1 could be explained by higher *Pm3b* expression levels in Pm3b#2 compared to Pm3b#1. However, Pm3b#1 and Pm3b#4 showed similar expression levels of *Pm3b* transcripts, but no pleiotropic effects were detected in Pm3b#1, indicating that not only expression levels but also position effects with subtle changes in expression over time and space might influence the phenotype (Brunner *et al*., 2011).

In the present study, we found that one of two lines transgenic for *Hv*‐*Ger4c*::*Ta*‐*Lr34res* analysed in detail (line 8) showed enhanced disease resistance without negative pleiotropic effects under glasshouse and near‐field conditions. This demonstrates that the *Ta*‐*Lr34res* gene can be used as a resistance source in barley, very similarly to wheat.

The only pathogen‐inducible barley promoter with demonstrated functionality in driving conditional expression of a transgene is *Hv*‐*Ger4c* (Himmelbach *et al*., 2010). Although we found this promoter to be highly useful in our work, it might be worth exploring additional inducible promoters for fine‐tuning expression levels. It has been shown that pathogen‐ and defence hormone‐inducible promoters can be functional in heterologous species, as shown for *Os*‐*PR10a* (induced by *Xanthomonas oryzae* pv *oryzae* and salicylic acid, SA), which was activated by SA in stably transformed *Arabidopsis* (Hwang *et al*., 2008). Together with the rapid accumulation of RNAseq data that allow a fast identification of pathogen‐inducible genes, there are an increasing number of candidate promoters that can be used for analysis of inducible expression.

In this study, two lines expressing *Ta*‐*Lr34res* under control of the *Hv*‐*Ger4c* promoter were selected for detailed analyses. There, we detected some basal expression of *Ta*‐*Lr34res* in the *Hv*‐*Ger4c*::*Ta*‐*Lr34res* lines and *Ta*‐*Lr34res* was specifically induced by the *Hv*‐*Ger4c* promoter after infection with powdery mildew, but not with barley leaf rust. Similarly, Himmelbach *et al*. (2010) measured a lower induction of *Hv*‐*Ger4c* promoter‐driven *GUS* expression after infection with barley leaf rust compared to powdery mildew. This could be due to the fact that barley powdery mildew infects epidermal cells, whereas barley leaf rust grows mainly in the mesophyll. Possibly, this indicates differences in inducibility of the promoter by pathogens in different tissues of barley. The fact that *Hv*‐*Ger4c*::*Ta*‐*Lr34res* lines showed resistance against barley leaf rust although an induction of *Ta*‐*Lr34res* expression was not confirmed indicates that the detected basal expression of *Ta*‐*Lr34res* was sufficient to mediate disease resistance.

The combination of different resistance genes is generally considered to be a successful strategy to enhance resistance. In barley, a considerable number of resistance genes have been described (Andersen *et al*., 2016; Seeholzer *et al*., 2010). The most well‐known and cloned major barley resistance genes that act efficiently as single genes are *Rpg1* and the *Mla* alleles (Brueggeman *et al*., 2002; Chelkowski *et al*., 2003). It has been shown that the use of quantitatively acting resistance genes can extend the life expectancy of *R* genes and the combination of these two types of resistance is a very promising approach to achieve durable resistance in agricultural ecosystems (Brun *et al*., 2010; McDonald, 2010). Thus, a combination of, e.g. *Mla* genes with the quantitatively acting *Ta*‐*Lr34res* might be an effective strategy to achieve durable resistance against mildew in barley. Another quantitatively acting gene in barley mildew resistance is the modulator of defence and cell death *Mlo* gene, which encodes a seven‐transmembrane domain protein and is a negative regulator of pathogen defence response. Homozygous loss‐of‐function mutant (*mlo*) barley plants show enhanced and durable resistance against barley powdery mildew (Jorgensen, 1992). So far, several mutant alleles (*mlo*) were described, which resulted in inhibited fungal growth (Buschges *et al*., 1997; Piffanelli *et al*., 2002).

The *Rpg1* gene encodes a protein with two tandem kinase domains and has been used in the northern parts of the USA and Canada to protect barley from stem rust for more than 65 years (Brueggeman *et al*., 2002). Therefore, *Rpg1* confers durable resistance, but in contrast to *Ta*‐*Lr34res* this is a resistance active specifically against stem rust. It would be interesting to cross the *Hv*‐*Ger4c*::*Ta*‐*Lr34res* lines with Morex, the donor of *Rpg1*, to test whether the *Rpg1/Ta*‐*Lr34res* gene combination would result in still improved resistance compared to the corresponding parental lines. Pyramiding different resistance genes or alleles seems promising to enlarge spectrum and durability of pathogen resistance. However, stacking of certain different *Pm3* alleles resulted in suppression of the *Pm3*‐mediated resistance response, indicating that combining of resistance genes and/or alleles is not always useful (Stirnweis *et al*., 2014).

Earlier studies demonstrated that *Ta*‐*Lr34res* is transferrable to different crop species and was shown to confer durable, partial resistance also in these heterologous species (Chauhan *et al*., 2015; Krattinger *et al*., 2016; Rinaldo *et al*., 2016; Risk *et al*., 2013; Sucher *et al*., 2016). Interestingly, the expression of *Ta*‐*Lr34res* was accompanied by negative pleiotropic effects, leading to increased fitness costs in barley and certain rice lines but not in durum wheat and maize. The data of this study and the results presented for barley by Chauhan *et al*. (2015) show a correlation of *Ta*‐*Lr34res* expression and the level of disease resistance as well as negative pleiotropic effects in barley. Similar results have been reported in rice (Krattinger *et al*., 2016). In all rice lines transgenic for *Ta*‐*Lr34res*, expression of the gene was controlled by its native promoter, and Krattinger *et al*. (2016) identified one low‐expressing transgenic rice line with no negative pleiotropic effects. This indicates that the genetic backgrounds of rice, maize and durum are all suitable to express *Ta*‐*Lr34res* under the control of its own promoter to give good resistance without fitness costs, at least in some carefully selected genotypes. In contrast, in barley no *Ta*‐*Lr34res* transgenic line under control of the native wheat promoter showed expression levels of *Ta*‐*Lr34res* which were low enough to avoid negative pleiotropic effects. The genetic background of barley seems not to be suitable to allow optimal *Ta*‐*Lr34res* expression levels when controlled by the native wheat promoter. Our results show that the negative pleiotropic effects of *Ta*‐*Lr34res* in barley can be overcome by induced expression of *Ta*‐*Lr34res* using the *Hv*‐*Ger4c* promoter. Therefore, barley plants transgenic for *Ta*‐*Lr34res* are potentially useful for agricultural applications. It seems rather straightforward to generate a large number of *Hv*‐*Ger4c*::*Ta*‐*Lr34res* lines or cross line 8 to elite barley cultivars and to select from them the lines with good resistance levels but no pleiotropic effects as determined under field conditions. Such lines would then represent ideal parents in breeding programmes.

## Experimental procedures

### Cloning of *Hv*‐*Ger4c* promoter upstream of genomic fragments of *Ta*‐*Lr34res* and stable transformation in barley

The Gateway binary vector p6UGLP∆SwaI containing the *Hv*‐*Ger4c* promoter was used as a source for the promoter sequence (Himmelbach *et al*., 2010). The native promoter of *Ta*‐*Lr34res* in constructs p6U:*Lr34*‐ResGenomic (Risk *et al*., 2013) was replaced by a two way cloning strategy. First the full‐length cDNA of *Ta*‐*Lr34res* was cloned in the p6UGLP∆SwaI by Gateway cloning method resulting in the p6UGLP∆SwaI:*Lr34res*cDNA plasmid. There is a unique *Nru*I restriction site within the very first exon of *Ta*‐*Lr34res,* and a *Mlu*I site is present just upstream of *Ta*‐*Lr34res* native promoter in p6U:*Lr34*‐ResGenomic. The fragment containing *Hv*‐*Ger4c* promoter and part of *Ta*‐*Lr34res* cDNA till the *Nru*I site was amplified with primers MluI‐proGLP‐Fwd (5′‐ACGCGTCTGCAGGAATTCGATCAGC‐3′) and NruI‐*Lr34*‐Rev (5′‐CGCTGTCGCGATGCCAATTCTAACTCGG‐3′) and cloned in PCR cloning vector pSCB (Agilent Technologies, Santa Clara (CA), USA) resulting in the pSCB:MluIProGLP‐*Lr34*NruI plasmid. After confirmation by sequencing, the promoter *Hv*‐*Ger4c* flanked by the *Mlu*I site and *Ta*‐*Lr34res Nru*I fragment was replaced in the original p6U:*Lr34*‐ResGenomic with *Mlu*I and *Nru*I restriction sites thereby exchanging the native *Ta*‐*Lr34res* promoter with pathogen‐inducible *Hv*‐*Ger4c* promoter.

Stable transformation of barley cv. Golden Promise was performed using *Agrobacterium*‐mediated stable transformation according (Hensel *et al*., 2009) with the exception that the callus‐forming media contained 1.25 mg/L CuSO_4_ pentahydrate and the *A. tumefaciens* strain AGL‐1 was used.

### Selection of transformants uniform for resistance to powdery mildew

T0 plants were grown to adult stage, and T1 grains were harvested. Two‐week‐old T1 seedlings were infected with powdery mildew isolate K1 and analysed macroscopically for *Ta*‐*Lr34res*‐mediated resistance (7 dpi). To do so, the barley line BG 9, transgenic for *Ta*‐*Lr34res* under the native promoter and its corresponding sister line BG 9 sib (described earlier, (Risk *et al*., 2013)) were used as positive and negative control, respectively. Resistant T1 progeny was then checked for presence of *Hv*‐*Ger4c*::*Ta*‐*Lr34res* on genomic DNA level by PCR and analysed for full‐length cDNA expression (Table S1). Finally, copy number was determined by Southern blot analysis.

### RNA extraction

Total RNA was extracted from third leaves using SV Total RNA Isolation system (Promega, Madison (WI), USA), and RNA integrity was checked by electrophoresis on a 1.2% agarose gel in 1x sodium‐borate buffer. For cDNA synthesis, RNA concentration was measured with a Nanodrop ND‐1000 spectrophotometer and 500 ng of total RNA was used for cDNA synthesis with the iScriptTM advanced cDNA synthesis kit (Bio Rad, Hercules (CA), USA).

### Detection of transgenic barley and determination of transgene copy number and full‐length cDNA amplification

Total genomic DNA was extracted from leaves using the CTAB method (Stein *et al*., 2001), and presence of *Ta*‐*Lr34res* was assessed using the marker csffr1 (Lagudah *et al*., 2009). Southern blot was performed by digesting 10 μg of genomic DNA with *EcoR*I and using a ^32^P‐labelled probe covering the *HPT* gene of the p6U vector as described previously (Risk *et al*., 2013). Segregants of the T1 generation negative in PCR were propagated to T2 and checked by Southern blot analysis. If absence of *Ta*‐*Lr34res* was confirmed, they were considered to be azygous sister lines (sib).

To check for full‐length cDNA expression, RNA was transcribed to cDNA using the M‐MLV reverse transcriptase according to the manufacturer's protocol (Invitrogen, Carlsbad (CA), USA) and a poly‐T (30) oligo. The 4319 bp amplicon was amplified using the Phusion^®^ High‐Fidelity DNA Polymerase (New Englands BioLabs, Ipswich (MA), USA) and a Touch‐down PCR protocol (1. 98 °C 3 min, 2. 98 °C 10 s, 3. 67 °C 20 s, 72° 2 min 50 s, 4. go 10× to 2. while reducing annealing temperature 1 °C per cycle, 5. 95 °C 10 s; 6. 55 °C 20 s, 7. 72 °C 2 min 50 s, go 25× to 5, 8. 72° 5 min). The primers used were FLC_F (5′GAGTACGGCTAGGCAATAGC3′) and FLC_RW (5′GGCAAGTAGCTATATCTGTAAC3′) whereby FLC stands for “full‐length cDNA”.

### Determination of *Ta*‐*Lr34res* gene expression by RT‐qPCR

Expression was determined by RT‐qPCR using a CFX96 or a CFX384 Touch Real‐time PCR machine (Bio Rad, Hercules (CA), USA). Reactions were set up with 5 μL KAPA SYBR fast qPCR mix (KAPA biosystems, Wilmington (MA), USA), *Ta*‐*Lr34res* primer (500 nm final concentration) (Risk *et al*., 2012) and 4 μL of 1:20 diluted cDNA per sample. *GAPDH* (Primers: *Gapdh*_Fw 5′CCGGGTTCCCACTGTGGAT3′ and *Gapdh*_Rw 5′TGACTAGCAACTCGGTGCGG3′; *E* = 102.0% *R* ^2^ = 0.977 Slope = 3.466 y‐int = 21.012) and *ADP* (Gimenez *et al*., 2011) were used as reference genes. For each run, four technical replicates were used and four to seven biological replicates were used in all experiments.

### Growth conditions and infection assays

Grains were sown in 96‐well Jiffy pots filled with soil (Einheitserde Profi Substrat, Einheitserde Werkverband e.v., Germany) and put over night at 4 °C in the dark for synchronized germination. Afterwards, plants were transferred to a growth chamber (16 h, 20 °C, light and 8 h, 17 °C, darkness) until indicated time points of the respective experiments. Seedling propagation was performed by repotting plants to 2 L pots (one plant per pot) and growing them in glasshouse at standard growth conditions.

The convertible glasshouse system was described in (Romeis *et al*., 2007). Its roof is automatically opened during dry and windless weather conditions. In addition, one side is permanently open which allows outdoor temperatures (Figure S5). Twenty plants each of lines BG 9, BG 9 sib, lines 8, 8 sib, 11 and 11 sib were sown on 8th of March 2016 in central cylinders (42.2 L) within big plastic containers filled with fresh farm soil which was taken freshly from a neighbouring field. Plants were grown until the five‐leaf stage and reduced to ten plants per central cylinder. Five cylinders were used per line. The surrounding area was planted with Golden Promise (GP) as buffering plants. Plastic containers were arranged in two blocks, and within each block each line was assigned randomly to a cylinder. The growth parameter measurements under glasshouse conditions were performed in 15 L pots instead of cylinders in a similar manner with the exception that due to a lack of space no plastic containers with surrounding buffering plants were grown.

Barley leaf rust infections were performed as described in Risk *et al*. (2012) using spore suspension of the barley leaf rust isolate BRG1.2.1 in 3M_Fluorinert_FC‐43 (3M, Switzerland). Rust infected plants were grown in a Conviron BDW80 chamber (Conviron, Canada) (16 h, 20 °C, light and 8 h, 17 °C, darkness). Mock infiltrations were also performed by spraying plants with FC‐43 without spores. Powdery mildew infection was performed by shaking the spores of isolate K1 from a pot containing approximately 25 infected plants (21 dpg) of 7 dpi barley cv. Golden Promise. Mock infections of the mildew experiments were performed by keeping a similar plant set under the same growth conditions without previous infection. Mildew infected plants were grown in a Sanyo MLR351 incubator (Sanyo, Japan) (16 h light, constantly 20 °C). Artificial powdery mildew infections in the convertible glasshouse were performed by potting two single plants (21 dpg, 14 dpi, infection performed in the laboratory) to the opposite edges of the surrounding area containing buffer plants at the time point when the plants in the cylinder reached the two leaf stage.

### Chitin measurement

Chitin measurement was performed as described in Ayliffe *et al*. (2013, 2014) with the following exceptions. Samples were diluted 1 : 10 for measurement; samples were treated for 20 min a steam cooker instead of autoclaving; the fluorometer Synergy H1 Hybrid 311 Reader (BioTek Instruments GmbH, Switzerland) was used for measuring. Four technical replicates were used for each sample. The readouts were analysed using the software Gen5, version 2.03.1 (BioTek Instruments GmbH, Switzerland). Chitin amounts were calculated using standard curves (Figure S3) generated by defined amounts of Chitin from shrimp shells (Sigma‐Aldrich, St. Louis (MO), USA).

### Statistical analysis

Differences in μg chitin per mg fresh weight and differences growth parameter values per plant across lines were tested using an ANOVA. If necessary, data were square‐root‐transformed to ensure normal distribution of residuals. When the normal distribution could not be reached, Kruskal–Wallis test was used. Multiple comparison *P*‐values were then computed using the post hoc Tukey–Kramer HSD or Kruskal–Nemenyi tests. Expression values were log_10_‐transformed to correct the exponential character of RT‐qPCR. Differences in expression were tested with an ANOVA, following the same procedure as described above. All statistical analyses were performed in R v.3.2.1. Critical *P*‐values were used for the decision for significance to create the letter codes. Critical *P*‐values, *P*‐values for all graphs shown and the transformation of raw data are given in Table S2.

## Supporting information


**Figure S1.** Southern blot of *Hv*‐*Ger4c*::*Ta*‐*Lr34res* barley lines.
**Figure S2.** Additional growth parameters to Figure 2.
**Figure S3.** Standard curves for chitin measurements shown in Figure 1.
**Figure S4.** Normalized relative expression of *Ta*‐*Lr34res* in plants grown in the convertible glasshouse under near‐field conditions.
**Figure S5.** The convertible glasshouse enables near‐field growth conditions.
**Table S1.** Phenotypical analysis of T1 progeny from different transformation events.
**Table S2.** *P*‐values, transformation of raw data and critical *P*‐values for statistical analysis.
