# Supplementary material for: Pathogen‐inducible Ta‐Lr34res expression in heterologous barley confers disease resistance without negative pleiotropic effects
Source: Plant Biotechnol J. 2017 Jul 11;16(1):245–53. doi: 10.1111/pbi.12765 (PMC5785347; doi:10.1111/pbi.12765)
Supplement: Supplementary file 1 — Figure S1. Southern blot of Hv‐Ger4c::Ta‐Lr34res barley lines. Figure S2. Additional growth parameters to Figure 2. Figure S3. Standard curves for chitin measurements shown in Figure 1. Figure S4. Normalized relative expression of Ta‐Lr34res in plants grown in the convertible glasshouse under near‐field conditions. Figure S5. The convertible glasshouse enables near‐field growth conditions. Table S1. Phenotypical analysis of T1 progeny from different transformation events. Table S2. P‐values, transformation of raw data and critical P‐values for statistical analysis. [file PBI-16-245-s001.pdf]

Transformant family T1	LTN at seedling stage	resistance	full length cDNA
2	yes	moderate	n.t.
3	yes	moderate	n.t.
4	yes	moderate to strong	n.t.
5	yes	moderate	+
6	no	no	n.t.
7	no	weak	n.t.
8	no	moderate to strong	+
9	no	moderate	+
10	no	moderate	+
11	weak	moderate to strong	+
12	yes	moderate to strong	+
13	no	weak	+
14	yes	moderate to strong	+
15	no	weak	+
16	no	no	-
17	no	weak	+
18	no	weak	+
19	no	weak	+
BG9	yes	strong	+

Supplemental Table 1. Phenotypical analysis of T1 progeny from different transformation events
Two week old T1 plants were assessed for the presence/absence of LTN and infected with the barley powdery mildew isolate K1. 7 dpi, plants were macroscopically analyzed for resistance by presence/absence of infection symptoms. Later, full-length cDNA expression was studied by amplifying the complete 4,319bp cDNA fragment using PCR. The T0 plant of transformation event 1 produced no grains and is therefore not listed. Progeny of transformation events 8 and 11 (indicated in yellow) were chosen for further analysis because these lines showed high resistance levels while developing no or a weak LTN phenotype at seedling stage. n.t. = not tested.

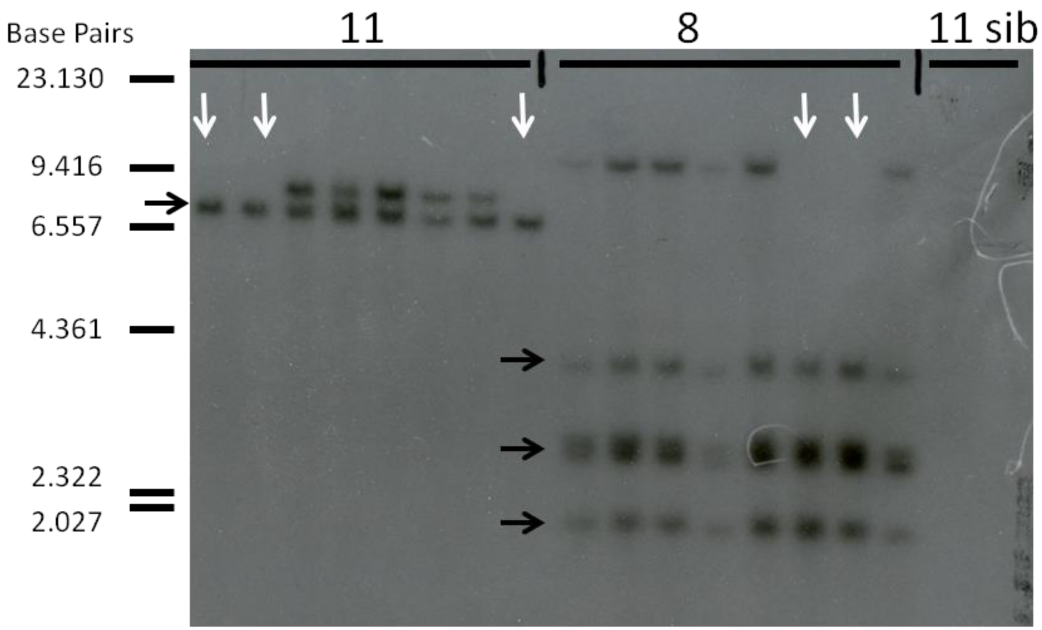

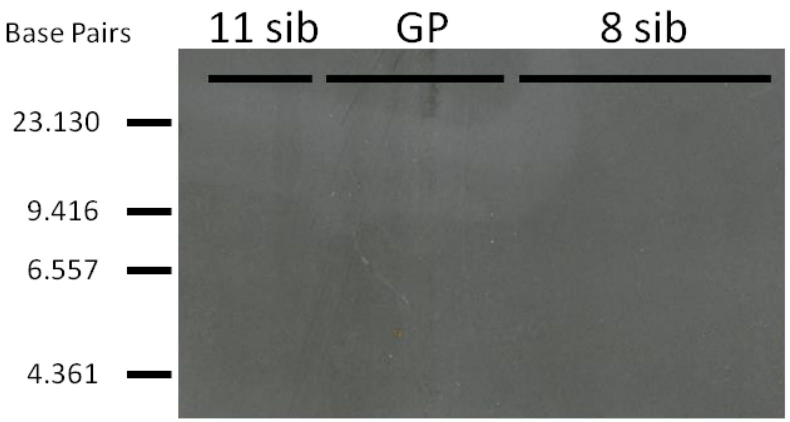

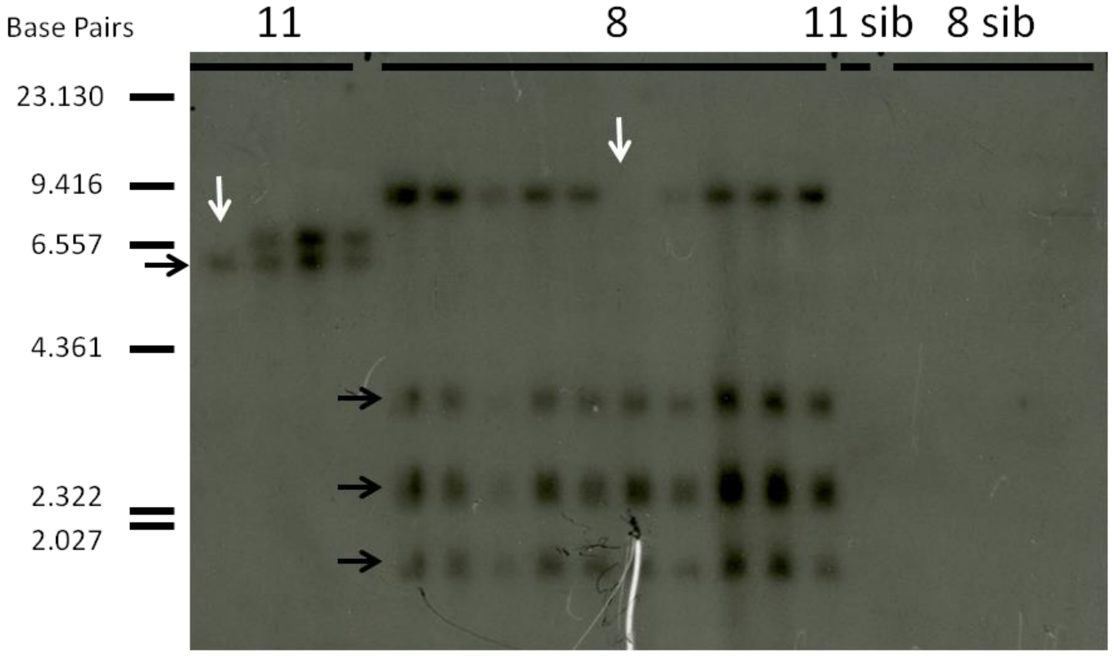

<u>Supplemental Figure 1.</u>
<u>Southern blot of *Hv-Ger4c*::*Ta-Lr34res* barley lines.</u>
10µg of genomic DNA were digested with *Eco*RI and probed with a ^{32}P-labeled probe covering the *HPT* gene of the p6U vector. Black arrows show non-segregating copies, white arrows indicate missing bands, indicating segregating copies.

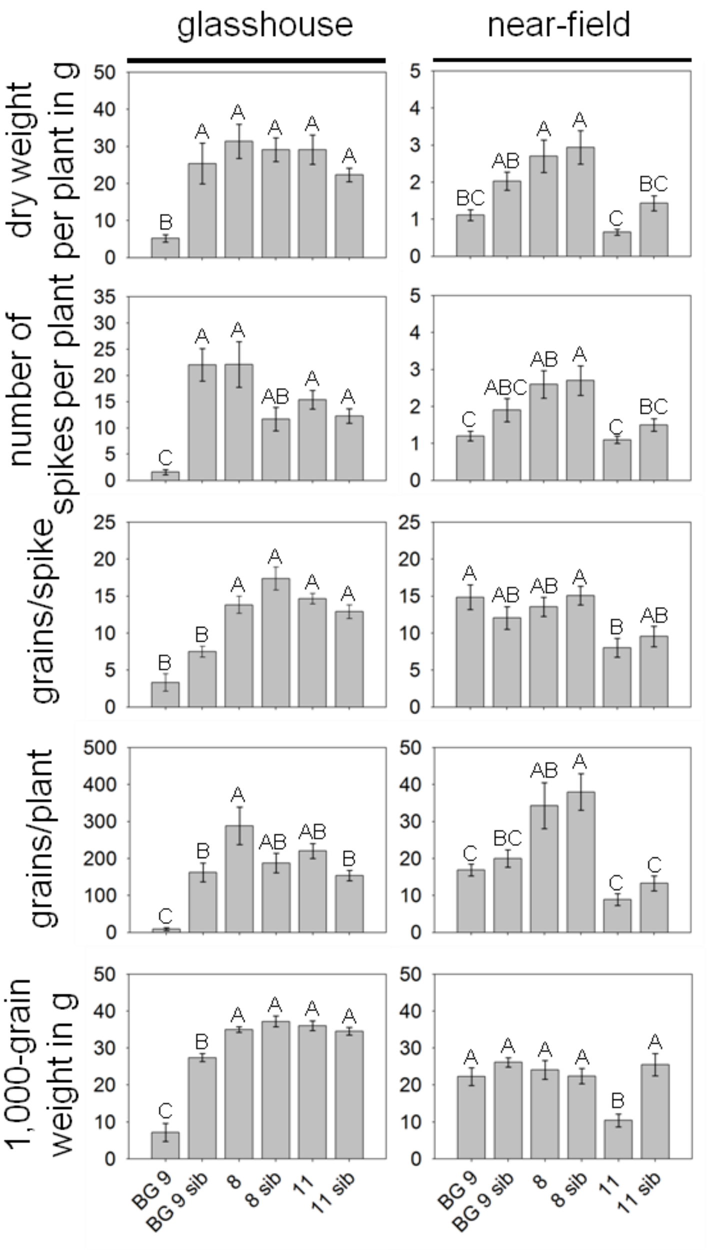

<u>Supplemental Figure 2.</u> <u>Additional growth parameters to Figure 2.</u>
For assessment of growth parameters, plants were grown until maturity, 10 individual plants were harvested growth parameters as indicated were determined per plant. Plants under standard glasshouse conditions were 138 days, plants under near-field conditions were 140 days old. Error bars indicate standard errors. Transformation of raw data for statistical analysis by Tukey-Kramer HSD test and relevant p-values are mentioned in Supplemental Table 2. Levels not connected by the same letter are significantly different.

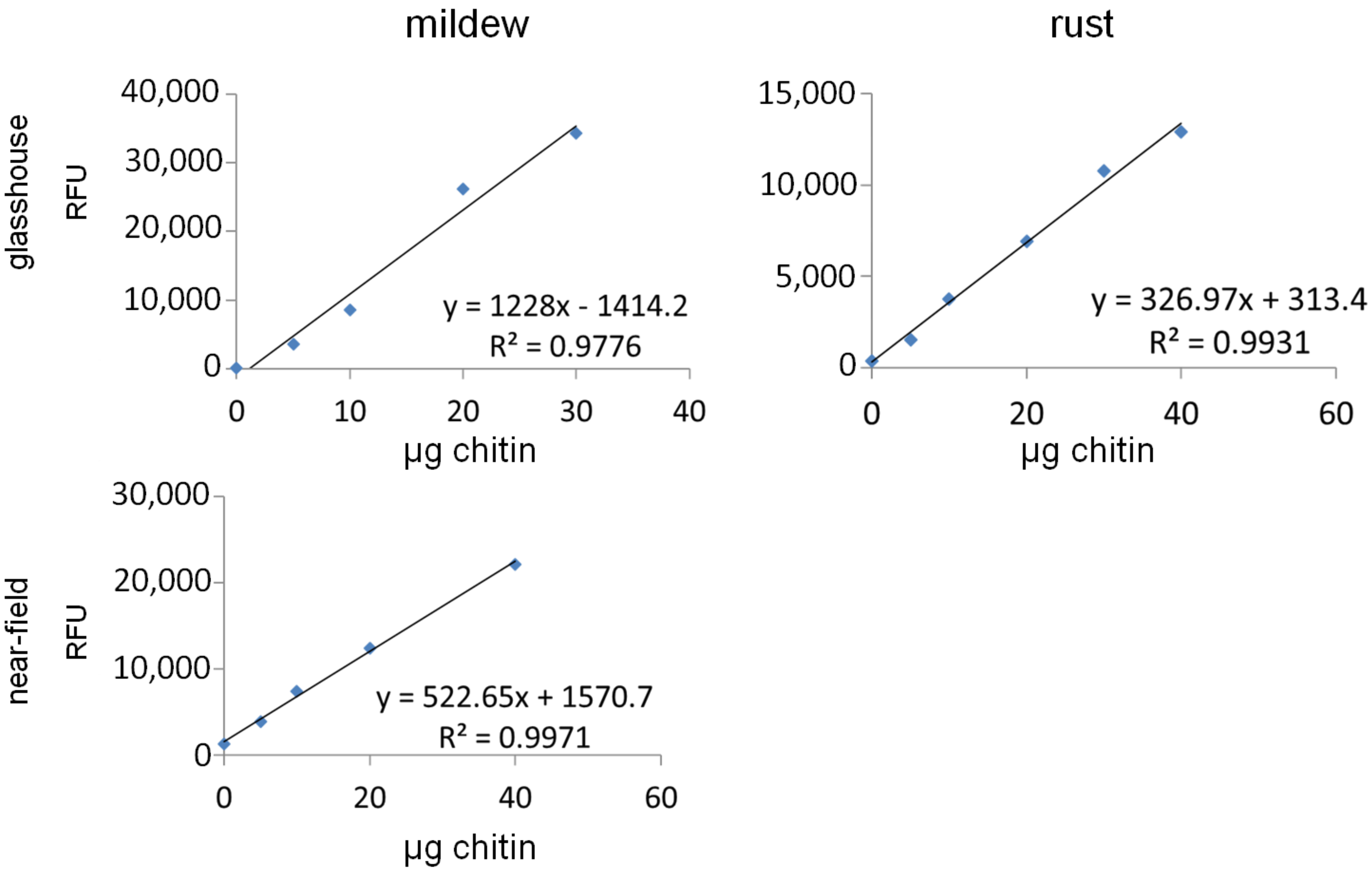

Supplemental Figure 3. Standard curves for chitin measurements shown in Figure 1.

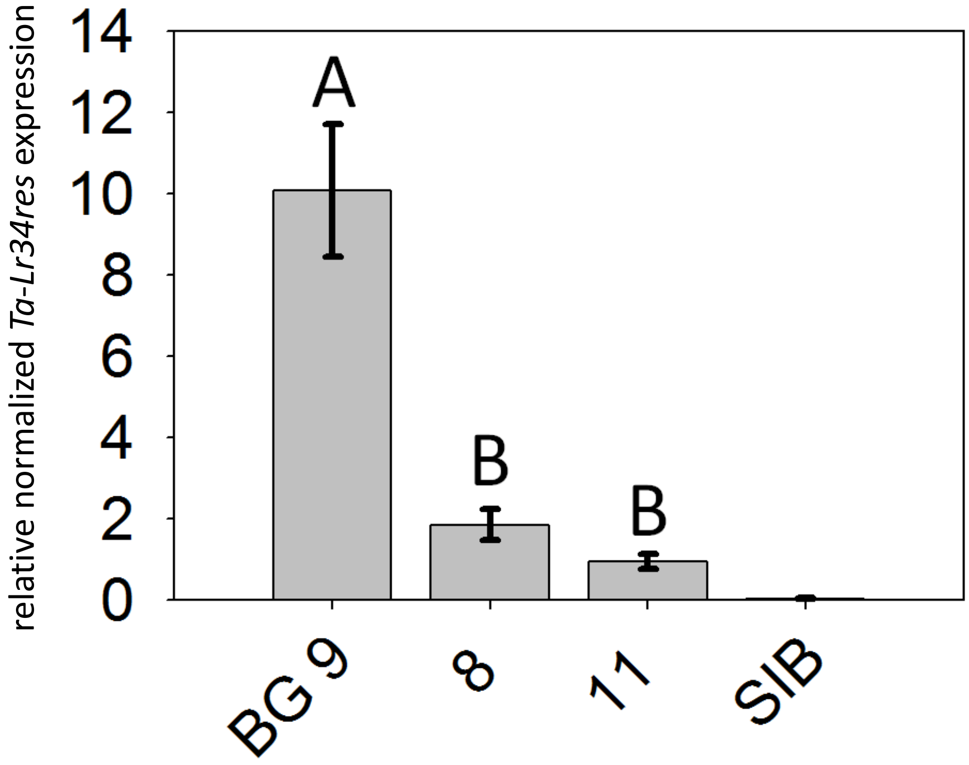

<u>Supplemental Figure 4. Normalized relative expression of *Ta-Lr34res* in plants grown in the convertible glasshouse under near-field conditions</u>

Third leaves of plants at five-leaf-stage were used for RNA extraction and expression analysis. Bars show the relative *Ta-Lr34res* expression normalized to *GAPDH* as an average of 7 biological replicates. Each replicate was measured four times (technical replicates). SIB represents the average of all sister lines representing the negative control. Error bars represent standard errors. Statistical analysis was done on $\log_{10}$-performed values using the all pairs Tukey-Kramer HSD test. Levels not connected by the same letter are significantly different. $p< 0.05$.

Figure 2 and supplemental Figure 2

A) Glasshouse

Trait	dryweight	tiller	seeds per tiller	seeds per plant	g seeds per plant	1000er
test	Anova	Anova	Anova	Anova	Anova	Anova
data transformation	SQRT	SQRT	no	SQRT	SQRT	no
overal p-value	F(5,54) = 14.4, p-value<0.0001	F(5,54) = 20.9, p-value<0.0001	F(5,54) = 23.31, p-value<0.0001	F(5,54) = 31.3, p-value<p-value<2e-16	F(5,54) = 41.6, p-value<2e-16	F(5,54) = 62.2, p-value<2e-16
	Post-hoc p-value	Post-hoc p-value	Post-hoc p-value	Post-hoc p-value	Post-hoc p-value	Post-hoc p-value
11 sib-11	0.75	0.92	0.86	0.41	0.23	0.98
8-11	1.00	0.62	0.99	0.74	0.77	1.00
8 sib-11	1.00	0.75	0.48	0.91	0.93	0.99
BG 9-11	0.00	<.0001	<.0001	<.0001	<.0001	<.0001
BG 9 sib-11	0.91	0.51	0.00	0.47	0.03	0.00
8-11 sib	0.55	0.12	0.99	0.02	0.01	1.00
8 sib-11 sib	0.73	1.00	0.05	0.94	0.79	0.79
BG 9-11 sib	0.00	0.00	0.00	<.0001	<.0001	<.0001
BG 9 sib-11 sib	1.00	0.08	0.01	1.00	0.93	0.01
8 sib-8	1.00	0.05	0.20	0.18	0.21	0.90
BG 9-8	<.0001	<.0001	0.00	<.0001	<.0001	<.0001
BG 9 sib-8	0.77	1.00	0.00	0.03	0.00	0.01
BG 9-8 sib	0.00	0.00	<.0001	<.0001	<.0001	<.0001
BG 9 sib-8 sib	0.91	0.03	0.00	0.97	0.23	0.00
BG 9 sib-BG 9	0.00	<.0001	0.08	<.0001	<.0001	<.0001

B) Near_field

Trait	dryweight	tiller	seeds per tiller	seeds per plant	g seeds per plant	1000er
test	Anova	Anova	Anova	Anova	Anova	Anova
data transformation	no	no	no	SQRT	SQRT	no
relevant p-value	F(5,54) = 9.3, p-value<0.0001	F(5,54) = 6.4, p-value<0.0001	F(5,54) = 4.1, p-value=0.003	F(5,54) =12.19, p-value<0.0001	F(5,54) = 13.1, p-value<0.0001	F(5,54) =6.6, p-value<0.0001
	Post-hoc p-value	Post-hoc p-value	Post-hoc p-value	Post-hoc p-value	Post-hoc p-value	Post-hoc p-value
11 sib-11	0.43	0.90	0.97	0.63	0.05	0.00
8-11	0.00	0.00	0.08	0.00	0.00	0.00
8 sib-11	0.00	0.00	0.01	0.00	0.00	0.01
BG 9-11	0.88	1.00	0.01	0.13	0.02	0.01
BG 9 sib-11	0.02	0.32	0.35	0.03	0.00	0.00
8-11 sib	0.04	0.07	0.36	0.00	0.01	1.00
8 sib-11 sib	0.01	0.03	0.08	0.00	0.00	0.92
BG 9-11 sib	0.97	0.97	0.10	0.92	1.00	0.91
BG 9 sib-11 sib	0.71	0.90	0.81	0.57	0.52	1.00
8 sib-8	0.99	1.00	0.97	0.97	0.99	0.99
BG 9-8	0.00	0.01	0.99	0.03	0.04	0.99
BG 9 sib-8	0.60	0.47	0.97	0.14	0.53	0.99
BG 9-8 sib	0.00	0.00	1.00	0.00	0.01	1.00
BG 9 sib-8 sib	0.27	0.32	0.66	0.02	0.22	0.85
BG 9 sib-BG 9	0.26	0.47	0.73	0.98	0.74	0.83

Supplemental Table 2. P-values, transformation of raw data and critical p-values for statistical analysis

Differences in µg chitin per mg fresh weight and differences growth parameter values per plant across lines were tested using an ANOVA. If necessary, data were square root transformed to ensure normal distribution of residuals. When the normal distribution could not be reached, Kruskal-Wallis test were used. Multiple comparison p-values were then computed using the Post-hoc Tukey-Kramer or Kruskal-Nemenyi tests. Expression values were $\log_{10}$-transformed to correct the exponential character of RT-qPCR. Differences in expression were tested with an ANOVA, following the same procedure as described above. All statistical analyses were performed in R v.3.2.1. Critical p-values were used for the decision for significance to create the letter codes.

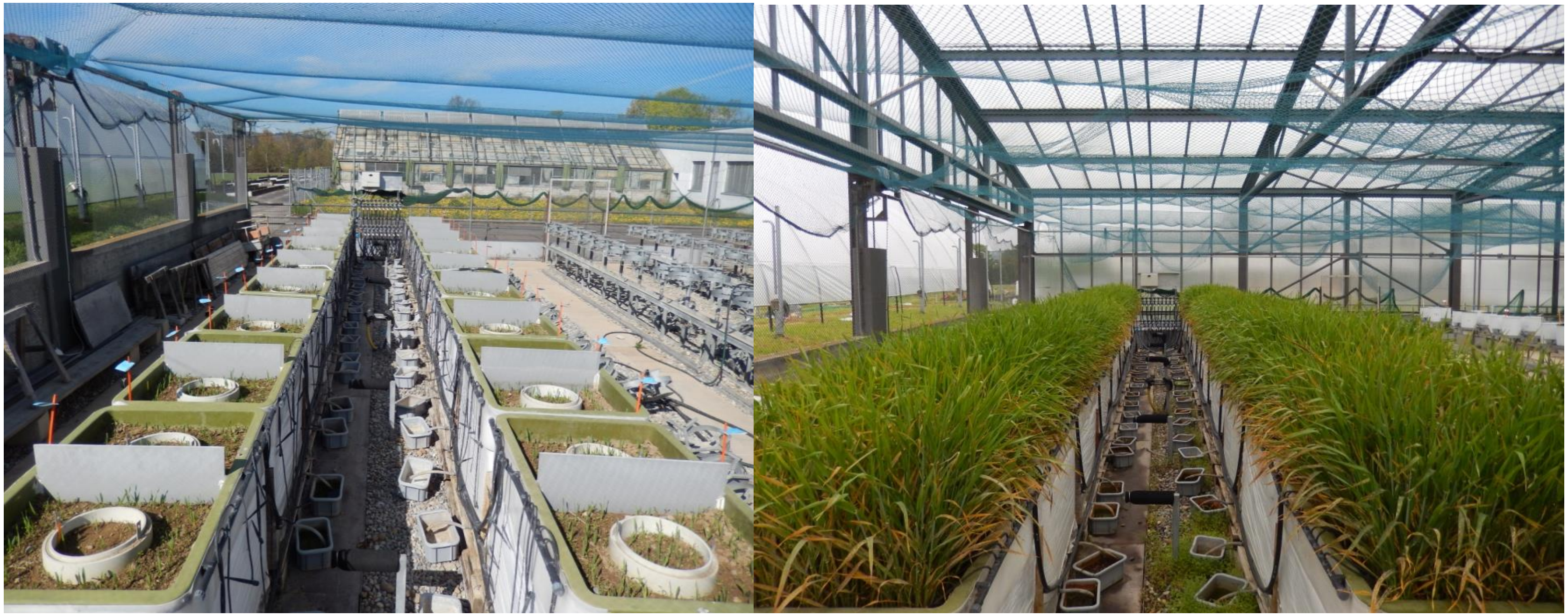

Supplemental Figure 5: The convertible glasshouse enables near-field growth conditions

Plants are grown in central cylinders surrounded by buffering plants. Under dry and windless conditions the roof is automatically opened (left). Furthermore, one side wall is permanently open to allow outdoor temperatures. The system is described in Romeis et al. (2007).
